# Supplementary material for: Effectiveness and Safety of Ayurvedic Medicines in Type 2 Diabetes Mellitus Management: A Systematic Review and Meta-Analysis
Source: Front Pharmacol. 2022 Jun 8;13:821810. doi: 10.3389/fphar.2022.821810 (PMC9213670; doi:10.3389/fphar.2022.821810)
Supplement: Supplementary file 1 [file DataSheet1.zip › Supplementary Material/Forest and Funnel Plots/Nigella sativa/HDL-C.pdf]

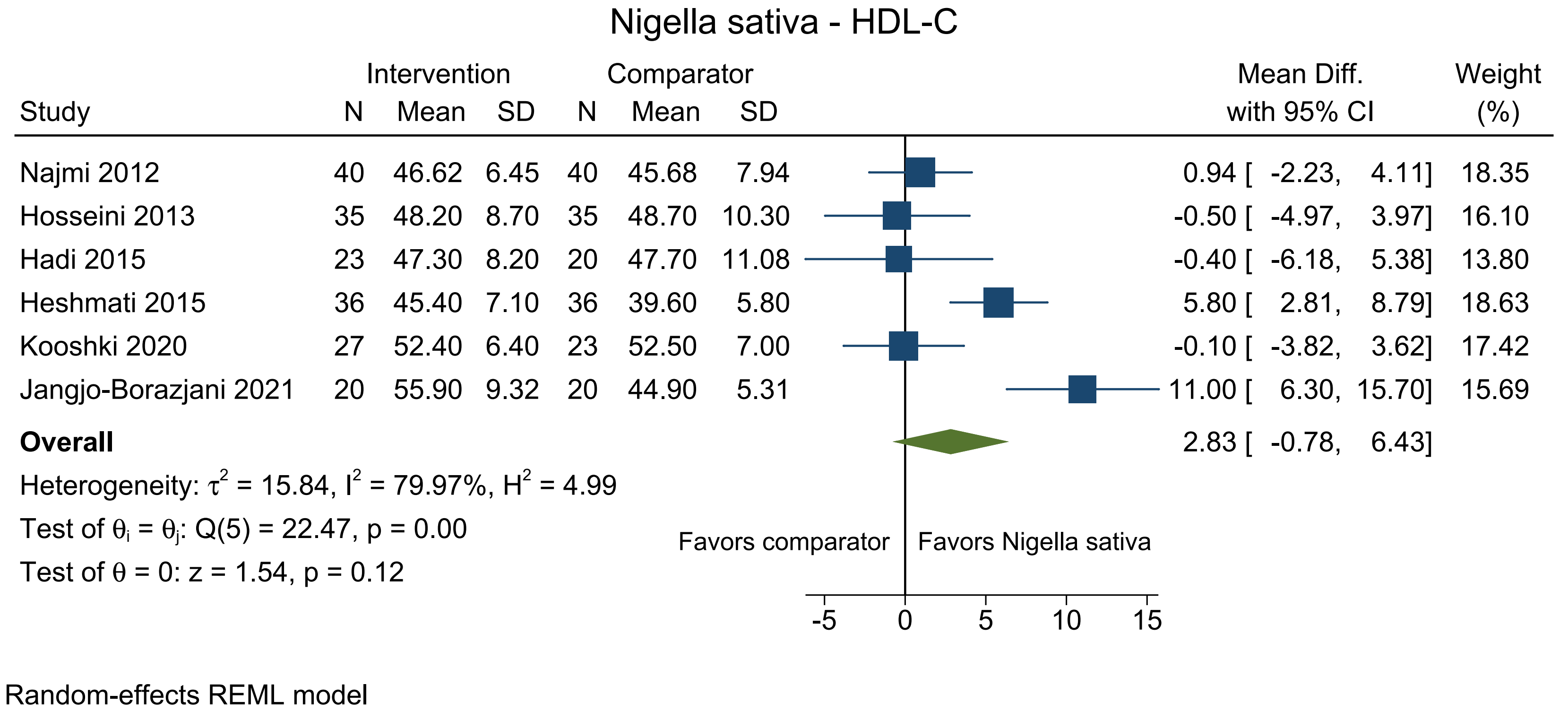
Nigella sativa - HDL-C
Intervention
Comparator
Mean Diff.
Weight
Study
N
Mean
SD
N
Mean
SD
with 95% CI
(%)
Najmi 2012 40 46.62 6.45 40 45.68 7.94 0.94 [-2.23, 4.11] 18.35
Hosseini 2013 35 48.20 8.70 35 48.70 10.30 -0.50 [-4.97, 3.97] 16.10
Hadi 2015 23 47.30 8.20 20 47.70 11.08 -0.40 [-6.18, 5.38] 13.80
Heshmati 2015 36 45.40 7.10 36 39.60 5.80 5.80 [2.81, 8.79] 18.63
Kooshki 2020 27 52.40 6.40 23 52.50 7.00 -0.10 [-3.82, 3.62] 17.42
Jangjo-Borazjani 2021 20 55.90 9.32 20 44.90 5.31 11.00 [6.30, 15.70] 15.69
Overall 2.83 [-0.78, 6.43]
Heterogeneity: τ^2 = 15.84, I^2 = 79.97%, H^2 = 4.99
Test of $\theta_i = \theta_j$: Q(5) = 22.47, p = 0.00
Test of θ = 0: z = 1.54, p = 0.12
Favors comparator
Favors Nigella sativa
-5
0
5
10
15
Random-effects REML model
